# GLD-4-Mediated Translational Activation Regulates the Size of the Proliferative Germ Cell Pool in the Adult *C. elegans* Germ Line

**DOI:** 10.1371/journal.pgen.1004647

**Published:** 2014-09-25

**Authors:** Sophia Millonigg, Ryuji Minasaki, Marco Nousch, Christian R. Eckmann

**Affiliations:** Max Planck Institute of Molecular Cell Biology and Genetics (MPI-CBG), Dresden, Germany; Harvard University, United States of America

## Abstract

To avoid organ dysfunction as a consequence of tissue diminution or tumorous growth, a tight balance between cell proliferation and differentiation is maintained in metazoans. However, cell-intrinsic gene expression mechanisms controlling adult tissue homeostasis remain poorly understood. By focusing on the adult *Caenorhabditis elegans* reproductive tissue, we show that translational activation of mRNAs is a fundamental mechanism to maintain tissue homeostasis. Our genetic experiments identified the Trf4/5-type cytoplasmic poly(A) polymerase (cytoPAP) GLD-4 and its enzymatic activator GLS-1 to perform a dual role in regulating the size of the proliferative zone. Consistent with a ubiquitous expression of GLD-4 cytoPAP in proliferative germ cells, its genetic activity is required to maintain a robust proliferative adult germ cell pool, presumably by regulating many mRNA targets encoding proliferation-promoting factors. Based on translational reporters and endogenous protein expression analyses, we found that *gld-4* activity promotes GLP-1/Notch receptor expression, an essential factor of continued germ cell proliferation. RNA-protein interaction assays documented also a physical association of the GLD-4/GLS-1 cytoPAP complex with *glp-1* mRNA, and ribosomal fractionation studies established that GLD-4 cytoPAP activity facilitates translational efficiency of *glp-1* mRNA. Moreover, we found that in proliferative cells the differentiation-promoting factor, GLD-2 cytoPAP, is translationally repressed by the stem cell factor and PUF-type RNA-binding protein, FBF. This suggests that cytoPAP-mediated translational activation of proliferation-promoting factors, paired with PUF-mediated translational repression of differentiation factors, forms a translational control circuit that expands the proliferative germ cell pool. Our additional genetic experiments uncovered that the GLD-4/GLS-1 cytoPAP complex promotes also differentiation, forming a redundant translational circuit with GLD-2 cytoPAP and the translational repressor GLD-1 to restrict proliferation. Together with previous findings, our combined data reveals two interconnected translational activation/repression circuitries of broadly conserved RNA regulators that maintain the balance between adult germ cell proliferation and differentiation.

## Author Summary

Throughout adulthood, animal tissue homeostasis requires adult stem cell activities. A tight balance between self-renewal and differentiation protects against tissue overgrowth or loss. This balance is strongly influenced by niche-mediated signaling pathways that primarily trigger a transcriptional response in stem cells to promote self-renewal/proliferation. However, the cell-intrinsic mechanisms that modulate signaling pathways to promote proliferation or differentiation are poorly understood. Recently, post-transcriptional mRNA regulation emerged in diverse germline stem cell systems as an important gene expression mechanism, primarily preventing the protein synthesis of factors that promote the switch to differentiation. In the adult *C. elegans* germ line, this study finds that the evolutionarily conserved cytoplasmic poly(A) polymerase, GLD-4, plays an crucial role in maintaining a healthy balance between proliferation and differentiation forces. This is in part due to translational activation of the mRNA that encodes the germ cell-expressed Notch signaling receptor, an essential regulator of proliferation. Moreover, GLD-4 activity is part of a redundant genetic network downstream of Notch that, together with several other conserved mRNA regulators, promotes differentiation onset. Given the widespread expression of these conserved RNA regulators in metazoans, cell fate balances that are reinforced by translational activation and repression circuitries may therefore be a general mechanism of adult tissue maintenance.

## Introduction

During development, tissues grow to form functional organs. In adulthood, animal tissues remain constant in size, in part, as a result of the dynamic balance between self-renewal/proliferation and differentiation. Perturbation of this balance affects tissue homeostasis and, consequently, compromises organ function. While excess proliferation contributes to tumorigenesis, a deficit in proliferation leads to tissue degeneration. Hence, tight regulatory mechanisms are in place to control the balance between self-renewal/proliferation and differentiation. One prevalent cell-extrinsic regulatory mechanism of stem cells to self-renew/proliferate is their dependency on supporting niche cells, which trigger established signal transduction pathways that primarily lead to changes at the transcriptional level. However, to fine-tune proper tissue homeostasis and to provide tight feedback controls, additional cell-intrinsic gene expression mechanisms are likely to exist.

In recent years, invertebrate germline tissues emerged as powerful *in vivo* models to investigate the balance between proliferation and differentiation. One influential paradigm is the adult “female” germ line of *C. elegans*, which depends on a single somatic niche cell and maintains a strict spatio-temporal organization of proliferating and differentiating cells [1]. Undifferentiated germ cells proliferate exclusively in the distal end of the germ line, termed the proliferative zone (PZ) [2], [3], [4]. The PZ is proposed to contain a distal pool of germline stem cell-like cells (GSCs) and a proximal pool of transit amplifying cells that gradually mature to start differentiation at a defined distance from the distal end [1], [5], termed the mitosis-to-meiosis boundary. Germ cells crossing this boundary enter meiotic prophase, which is here defined as differentiation onset [2], [6], [7]. Germline proliferation relies on the Notch signaling pathway that is instructed by the somatic distal tip cell (DTC) [8], [9], [10]. Consistent with its continuous requirement for germ cell proliferation in the adult, the inactivation of Notch signaling leads to progressive loss of GSCs, due to differentiation of all germ cells [9]. Conversely, constitutive activation of Notch results in the expansion of the proliferative GSC pool at the expense of differentiation [11], [12]. In agreement with this, germ cells in the PZ express the Notch receptor GLP-1, while differentiating cells lose GLP-1 expression [13]. Hence, Notch-mediated transcriptional regulation of mitotic fate-promoting genes is suggested to directly maintain the proliferative fate [14], [15], [16]. However, germ cell-intrinsic mechanisms that promote niche-mediated germ cell proliferation are still widely unknown.

In nematodes and flies, germ cells also utilize conserved translational repressors to promote the undifferentiated state [17]. In *C. elegans*, two nearly identical translational repressors of the PUF RNA-binding protein family, FBF-1 and FBF-2, jointly referred to as FBF, are essential for adult GSCs [18]. FBF recognizes specific sequence elements (FBEs) in its mRNA targets and, by translationally repressing numerous meiosis-promoting genes, FBF is critical for maintaining the undifferentiated, proliferative state [7], [18], [19], [20]. Moreover, the *fbf-2* locus is a proposed target of Notch-mediated regulation [14], [21], thus linking transcriptional activation with translational repression, the two dominant mechanisms used for sustained germ cell proliferation in different organisms [1].

Across species, differentiation onset of germ cells depends on translational control [17]. In nematodes, the STAR-type RNA-binding protein, GLD-1, inhibits GLP-1 protein accumulation [22], [23] and recognizes *glp-1* mRNA by three GLD-1-binding motifs (GBMs) present in its 3′UTR [24], [25]. Meiotic prophase entry also requires the Nanos protein family member, NOS-3, a presumed translational repressor of yet unknown mitosis-promoting genes [21]. However, in the absence of GLD-1, NOS-3, or both, germ cells enter meiotic prophase in the adult [7], [26]. The cytoplasmic poly(A) polymerase (cytoPAP) complex GLD-2/GLD-3 is a proposed translational activator of meiosis-promoting mRNAs, envisioned to extend their poly(A) tail lengths. GLD-2 is a non-canonical nucleotidyltransferase, stimulated by the Bicaudal-C family member, GLD-3 [27], [28]. However, in the absence of GLD-2, GLD-3, or both, the PZ is expanded but meiosis is still initiated [7]. This complexity highlights that differentiation onset is in general a multi-pathway-regulated process [17].

In the current model of the core genetic network underlying differentiation onset in *C. elegans*, the four meiosis-promoting RNA regulators act in two parallel pathways. The two translational repressors (*gld-1* and *nos-3*) form the first pathway; the two translational activators (*gld-2* and *gld-3*) form the second pathway. This genetic redundancy is most apparent in germ cells that lack GLD-3 and NOS-3, as they do not enter meiotic prophase and continue to proliferate [7]. Importantly, tumorous proliferation of *gld-3 nos-3* double mutant germ cells is independent of Notch signaling and dependent on cyclin E activity [4], [7]. Intriguingly, germ cells lacking GLD-2 and NOS-3 are able to start meiotic prophase [6], [7]. This suggests that the current pathway assignments are too simplistic and emphasizes that more meiosis-promoting regulators must exist [4], [6], [7]. Especially, commitment to female meiotic progression provides precedence for redundant translational activation activities in *C. elegans*. Here, in addition to GLD-2 cytoPAP-mediated GLD-1 expression [29], the GLD-4/GLS-1 cytoPAP complex has been identified to translationally activate *gld-1* mRNA [30]. As a non-canonical poly(A) polymerase, GLD-4 is most similar to the conserved group of Trf4/5-type RNA modifiers that regulate RNA stability in the nucleus [30], [31], [32]. However, GLD-4 poly(A) polymerase is cytoplasmic and requires for its functions the nematode-specific protein, GLS-1 [30], [33]. In the absence of GLD-2, the GLD-4/GLS-1 cytoPAP complex is essential for female meiotic progression into pachytene [30].

In this study, we report that the GLD-4/GLS-1 cytoPAP complex has a dual role in regulating the balance between proliferation and differentiation. We find that the GLD-4/GLS-1 cytoPAP complex is crucial to maintain germ cell proliferation in the adult, in part by promoting robust translation of *glp-1* mRNA. Moreover, to ensure that meiosis-promoting factors are inefficiently translated, GLD-2 cytoPAP levels are kept low in the GSC pool by FBF-mediated translational repression. Lastly, we also find that GLD-4/GLS-1 cytoPAP promotes meiotic prophase entry, in parallel to GLD-2 cytoPAP and independently of Notch. Our data suggest that two translational feedback loops limit the size of the proliferative germ cell pool and maintain a healthy balance of germ cell proliferation and differentiation in the adult germ line.

## Results

### GLD-4/GLS-1 cytoPAP activity maintains the size of the proliferative zone

**Figure 1:**
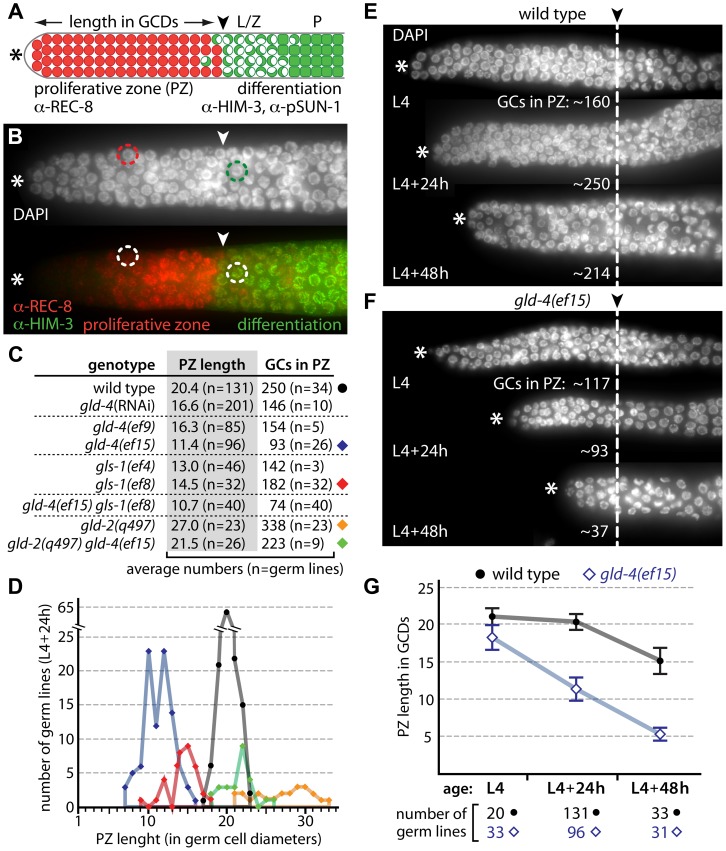
*gld-4* and *gld-2* have opposing functions in regulating the balance between proliferation and differentiation. (A) Diagram of the distal end of an adult germ line (not to scale). Germ cells divide in the proliferative zone (PZ; red circles) and express REC-8 prior to meiotic prophase entry. Germ cells in meiotic prophase (green) express HIM-3 and pSUN-1; leptotene/zygotene (L/Z; half-filled circles) and pachytene (P; squares). In adulthood, a mitosis-to-meiosis boundary (arrowhead) is maintained at a defined distance from the distal tip (asterisk). GCDs, germ cell diameters. (B) Corresponding immunofluorescence micrograph of a distal gonad, illustrating a typical PZ nucleus (red circle) and a crescent-shaped meiotic prophase nucleus (green circle). The mitosis-to-meiosis boundary is apparent from REC-8 and HIM-3 expression. (C,D) Proliferative zone measurements of cytoPAP mutant gonads reveal germ cell (GC) number differences in young adults (L4+24 h). Circle and diamonds to the right side of the table in (C) correspond to the data shown in (D). (E,F) Age-dependent PZ changes are enhanced in *gld-4* mutants. Dashed line, mitosis-to-meiosis boundary. (G) Quantification of PZ length changes of E and F over time. Error bars, standard deviations.

On average, the PZ of adult germ lines extends from the first germ cell row at the distal end further proximally to row 20, where germ cells start differentiation by entering meiotic prophase (Figure 1A,B). In wild type, the PZ is populated by about 225–250 germ cells (Figure 1C). Since there are no molecular markers for subpopulations of cells in the PZ, like stem cells, transit amplifying cells and cells in pre-meiotic S-phase, the start of meiotic prophase is commonly defined as the onset of differentiation [1], [34]. Differentiation is revealed by the germ cells' specific nuclear architecture and chromatin morphology, the combinatorial expression and localization of the meiotic cohesin REC-8, the synaptonemal protein HIM-3, and phosphorylated nuclear envelope protein pSUN-1 [34] (Figure 1A,B).

Germ cells in single mutants of meiosis-promoting genes (*i.e. gld-1, nos-3, gld-2, gld-3*) initiate meiotic prophase [7]. However, shifts in the position of the mitosis-to-meiosis boundary suggest a role in proliferation or differentiation. For example, in *gld-2* single mutants, the PZ is extended and contains more germ cells than wild type [7] (Figure 1C,D), consistent with *gld-2*'s function in promoting meiotic entry [35]. We found that *gld-4* and *gls-1* single mutants have smaller PZs with fewer germ cells (Figure 1C,D). The strength of the reduction appears to correlate with the reported allelic strengths of the individual mutations [30], [33] (Figure 1C). As the PZ of *gld-4 gls-1* double mutants is similarly reduced (Figure 1C), these results argue for a common role of *gld-4* and *gls-1* in promoting mitosis. The PZ of the *gld-2 gld-4* double mutant is similar to wild type in size and germ cell number (Figure 1C,D). Together, these results suggest that *gld-2* and *gld-4* have independent and opposing roles to set the mitosis-to-meiosis boundary in adults.

The PZ expands during larval development and is maintained during adulthood [8]. We measured the size of the PZ at the last larval stage (L4), and 24 hours (h), and 48 h later in young adults (Figure 1E–G). The difference between wild type and *gld-4* is the smallest in L4 and greatest during adulthood, due to a large relative shrinkage of the PZ in *gld-4* young adults (Figure 1E–G). Therefore, *gld-4* activity is primarily important for the maintenance but not establishment of the PZ during early adulthood.

### Expression of a *glp-1* mRNA translational reporter depends on GLD-4/GLS-1, but not on GLD-2, cytoPAP

The documented presence of GLD-4 [30] and GLS-1 [33] in the distal end of the germ line and the single mutant phenotypes argue for a role of *gld-4* and *gls-1* in promoting germ cell proliferation. CytoPAPs are envisioned to regulate poly(A) tail metabolism of target mRNAs in a positive manner [31]. Biochemically, cytoPAPs elongate poly(A) tails, which in turn stabilize mRNAs and enhance their translation. We hypothesized that GLD-4 targets mRNAs encoding proteins important for proliferation in the PZ. An obvious, but not exclusive, candidate for this regulation is the Notch receptor-encoding *glp-1* mRNA.

**Figure 2:**
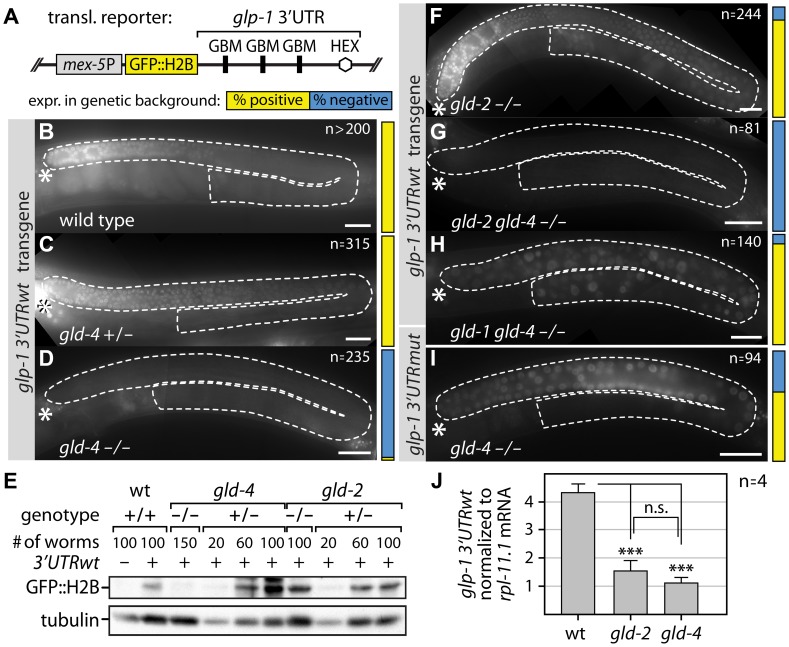
GLD-4 affects *glp-1* 3′UTR GFP reporter mRNA translation. (A) Schematic representation of the *glp-1* 3′UTR reporter transgene. Germline expression of a GFP-Histone 2B-fusion product (GFP::H2B) is driven by the *mex-5* promoter (*mex-5P*). GBM, GLD-1-binding motif; HEX, 3′end formation sequence. (B–D, F–I) Micrographs of adult gonads, expressing a GFP::H2B translational *glp-1* 3′UTR reporter in wild-type, heterozygous (+/−), or homozygous (−/−) mutant backgrounds, reveal *gld-4*-dependent reporter protein expression. Transgenic animals carry either a wild type (B–D, F–H) or a GBM-mutant (I) *glp-1* 3′UTR. The penetrance of analyzed germ lines (n) expressing the respective translational reporter is indicated by a color-coded bar (described in A) next to each micrograph. Dashed white lines mark gonads as assessed by DIC microscopy; asterisk, distal tip. Scale bars: 25 µm. (E) Quantification of GFP::H2B protein expression of the *glp-1* 3′UTRwt translational reporter in given genotypes by α-GFP immunoblotting; tubulin serves as loading control. #, number of animals per lane. (J) Quantification of *glp-1* 3′UTR reporter mRNA levels by RT-qPCR normalized to *rpl-11.1* mRNA. Endogenous *glp-1* mRNA levels were similar among all three genotypes. Error bars are standard error of the mean (SEM). ***, p<0.001; n.s., not significant (Student's t-test).

Notch expression is regulated at multiple levels in *C. elegans* [13], [23], [36]. To uncouple mRNA regulation from protein regulation, we used a translational reporter of GFP::H2B under the control of the *glp-1* 3′UTR [25] (Figure 2). The *glp-1* 3′UTR reporter is driven by a ubiquitous germ cell-specific promoter and encodes a translational fusion product of GFP and histone 2B (Figure 2A). This nuclear GFP signal reflects GLD-1-mediated regulation of the *glp-1* mRNA [25].

In a wild-type background, reporter GFP expression is present in all animals analyzed and its pattern is similar to endogenous GLP-1 protein expression [13], [25] (Figure 2B). To assess whether reporter GFP expression is under the influence of GLD-4 cytoPAP activity, we crossed the *glp-1* 3′UTR reporter locus into the strong loss-of-function *gld-4(ef15)* mutant background (Figure 2C,D). To control for unexpected genetic background influences, we compared heterozygous and homozygous *gld-4* siblings from the progeny of a heterozygous mother (see Materials and Methods). In the *gld-4* heterozygous mutant, reporter GFP expression is similar to a wild-type background (compare Figure 2B with C). Strikingly, upon *gld-4* removal, reporter expression was undetectable in almost all germ lines (Figure 2D). Consistent with a reduction in the GFP signal, we also observed lower GFP protein amounts by immunoblotting. When comparing *gld-4* animals to wild-type background, we observed a reduction of >80% in protein abundance (Figure 2E). These results imply that the expression of the *glp-1* 3′UTR reporter depends on *gld-4* cytoPAP activity.

GLS-1 and GLD-4 function together in meiotic progression [30], and in promoting differentiation onset (Figure 1C). Similar to the *gld-4* mutant, reporter GFP expression is undetectable in most *gls-1(ef8)* mutant germ lines (∼87%, n = 220), suggesting that *gls-1* promotes *glp-1* 3′UTR reporter expression similar to *gld-4* activity.

To investigate whether *gld-4* is the only known cytoPAP regulating reporter expression, we assessed GFP expression in *gld-2* mutants and detected it in almost all germ lines (Figure 2F). Moreover, the amounts of GFP reach wild-type protein levels and are similar between *gld-2* homozygous and heterozygous mutants (Figure 2E). Importantly, reporter GFP expression is still dependent on *gld-4* activity in the *gld-2* mutant background, as its expression is undetectable in all *gld-2 gld-4* homozygous double mutants (Figure 2G). These results suggest that *glp-1* 3′UTR reporter expression is largely independent of *gld-2* cytoPAP activity, and specifically dependent on *gld-4* cytoPAP activity.

### *glp-1* translational reporter expression is post-transcriptionally controlled

To further investigate at which level GLD-4 cytoPAP may regulate *glp-1* 3′UTR reporter expression, we made use of GLD-1, a known translational repressor of *glp-1* mRNA [23]. In *gld-1* single mutants, reporter GFP is expressed in the PZ and in differentiating germ cells (100%, n = 140). To test, whether loss of GLD-1 would de-repress reporter GFP expression in the *gld-4* mutant, we analyzed GFP::H2B expression in the *gld-1 gld-4* double mutant background. Most germ lines weakly express GFP when compared to *gld-4* mutants (compare Figure 2H with 2D). A similar weak de-repression is observed in *gld-4* mutant germ lines that contain mutated GLD-1-binding site reporter mRNAs (*glp-1* 3′UTR mut) (Figure 2I). Taken together, these results confirm that the *glp-1* 3′UTR reporter can be translated in a *gld-4* mutant background and that expression of the *glp-1* 3′UTR reporter is partly dependent on the GLD-4 cytoPAP even when GLD-1-mediated repression is removed.

Several mechanisms may account for reduced *glp-1* 3′UTR reporter expression in the absence of *gld-4*. To confirm that the effects on GFP::H2B expression are due to translational and not transcriptional regulation of the *glp-1* 3′UTR reporter, we examined the mRNA levels of the wild-type *glp-1* 3′UTR reporter by RT-qPCR (Figure 2J). Compared to wild type, we noticed a reduction of ∼4-fold in both *gld-4* and *gld-2* mutant backgrounds (Figure 2J), suggesting that *glp-1* 3′UTR reporter mRNA is less abundant in either cytoPAP mutant. Importantly, the *glp-1* 3′UTR reporter mRNA levels are similar to each other in both cytoPAP homozygous mutants, yet they give rise to different amounts of reporter protein (compare Figure 2D with 2F, and Figure 2E). Hence, we conclude that the major reduction in reporter GFP expression in *gld-4* mutants is primarily at the translational and not at the transcriptional level.

### Endogenous GLP-1 protein expression depends on GLD-4 cytoPAP activity

**Figure 3:**
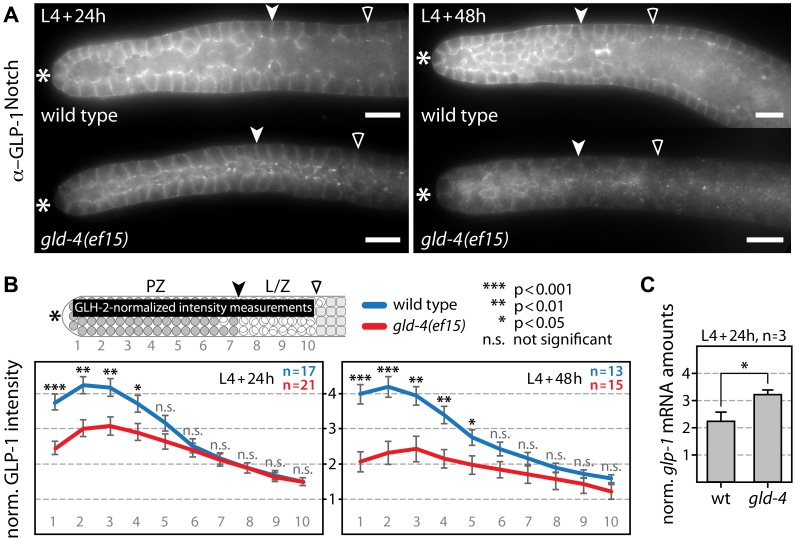
GLD-4 promotes endogenous GLP-1 expression. (A) Median images of extruded gonads stained with α-GLP-1 antibodies in given genetic backgrounds and at two developmental time points. Asterisk, distal tip; arrowhead, mitosis-to-meiosis boundary; empty carat, beginning of pachytene. Scale bars: 10 µm. (B) Quantification of the distal region of immunostained germ lines (n) from A. The α-GLP-1 fluorescent signal was normalized to α-GLH-2 signal. The values of the y-axis represent arbitrary units. Error bars are SEM; p values from Student's t-test. The top scheme indicates the area used for intensity measurements in the distal germ line (black bar). PZ, proliferative zone; L/Z, leptotene/zygotene; other label as in A. (C) Quantification of endogenous *glp-1* mRNA levels by RT-qPCR normalized to *rpl-11.1* mRNA. Error bars are SEM. *, p<0.05 (Student's t-test).

To further investigate whether endogenous GLP-1 protein expression is one likely candidate of *gld-4*-mediated regulation, we measured GLP-1 protein expression in *gld-4* mutants and compared it to wild type (Figure 3). By quantifying GLP-1 intensities in distal germ lines of L4+24 h and L4+48 h animals, we observed a significant decrease in the *gld-4* mutant background in the PZ over time (Figure 3A,B). When we measured endogenous *glp-1* mRNA levels in L4+24 h animals we observed a mild increase in *gld-4(ef15)* mutants compared to wild type (Figure 3C). Together these observations suggest that *gld-4* promotes GLP-1 expression post-transcriptionally.

**Figure 4:**
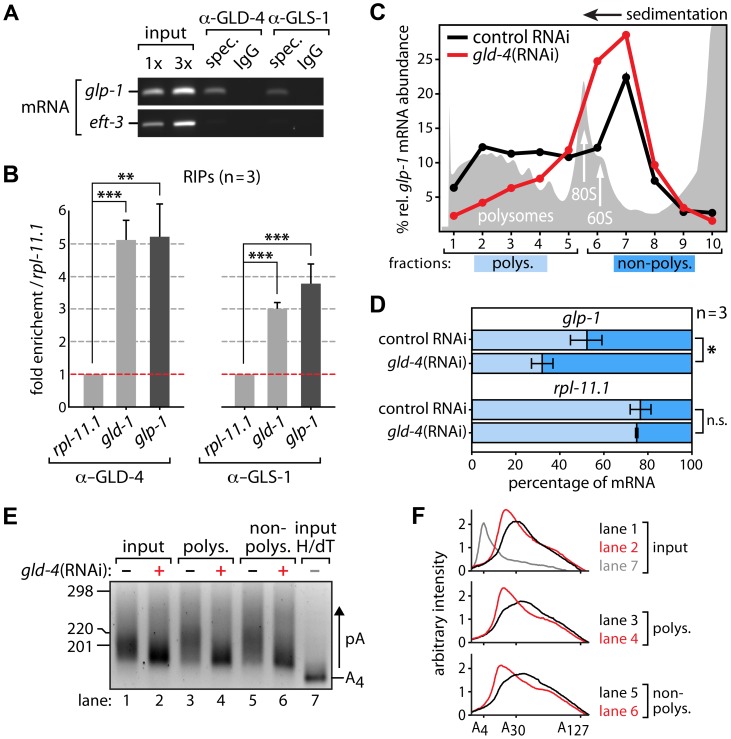
*glp-1* mRNA associates with GLD-4 and is a likely target of poly(A) tail extension and translational activation. (A,B) RNA-coimmunoprecipitation experiments (RIPs) of GLD-4 and GLS-1 proteins specifically enrich *glp-1* mRNA and the positive control *gld-1* mRNA. *eft-3* and *rpl-11.1* mRNA served as negative controls. (A) A representative ethidium bromide-stained agarose gel of semiquantitative RT-PCR products from three independent biological replicates. (B) Quantitative RT-PCR measurements of three additional RIPs. Error bars are SEM. ***, p<0.001; **, p<0.01; n.s., not significant (Student's t-test). (C,D) Translational efficiency of *glp-1* mRNA depends on *gld-4* activity. The data are representative of three independent biological experiments. (C) Polysome gradient. Top is to the right; grey peaks represent optical density read of 258 nm; the peaks of the large ribosomal subunit (60S), monosomes (80S), and polysomes are indicated. Relative *glp-1* mRNA levels are lower in polysome fractions of *gld-4*(RNAi) as measured by RT-qPCR. (D) Quantification and comparison of *glp-1* mRNA in pooled polysomal (polys.) and non-polysomal (non-polys.) fractions. Each measurement was normalized to an internal spike-in control (see Materials and Methods). Error bars are SEM. *, p<0.05; n.s., not significant (Student's t-test). (E,F) poly(A) tails of *glp-1* mRNA are reduced upon *gld-4*(RNAi). (E) Representative PAT assay (n = 2) of the *glp-1* mRNA material from (C) and the gradient input material. Nucleotide size marker to the left. Lane 7 reflects a 3′UTR with a strongly reduced poly(A) tail (pA) after RNAase H and oligo dT treatment (H/dT). (F) Line scans of PAT assay from (E).

A prerequisite for GLD-4/GLS-1-mediated *glp-1* mRNA regulation is that they form an mRNP complex. To test for a possible association of GLD-4 and GLS-1 with *glp-1* mRNA, we performed several RNA co-immunoprecipitation (RIP) experiments, using GLD-4-specific, GLS-1-specific, and non-specific antibodies. Subsequent RT-PCR (Figure 4A) and RT-qPCR (Figure 4B) analysis of different RIP experiments revealed a specific enrichment of endogenous *glp-1* mRNA, which is similar to the positive control, *gld-1* mRNA (Figure 4B). These results demonstrate an association of GLD-4/GLS-1 cytoPAP complex with endogenous *glp-1* mRNA and establish a potential physical link for *glp-1* mRNA translational regulation.

Cytoplasmic polyadenylation affects RNA stability and translational efficiency [37]. To test whether ribosomal engagement of the endogenous *glp-1* mRNA requires GLD-4 cytoPAP, we performed sucrose gradient sedimentation experiments. In theory, the more ribosomes are attached to an mRNA, the further the mRNA migrates into the gradient during ultra centrifugation. Therefore, efficiently translated mRNAs will be in the heavier, polysome fractions of the gradient, while poorly or non-translated mRNAs tend to sediment to lighter, non-polysomal fractions. Due to the large amounts of material needed, we compared control RNAi and *gld-4*(RNAi) knockdown worms (Figure 4C), knowing that *gld-4*(RNAi) efficacy is less robust than using mutants. In extracts of wild type and control RNAi (Figure 4C), about 50% of the endogenous *glp-1* mRNA resides in the polysome fraction, suggesting that half of the *glp-1* mRNA population is actively translated, consistent with the known germline and embryonic translational repression of *glp-1* mRNA [23], [38]. Upon knockdown of *gld-4*, but not in control RNAi, we observed a shift of *glp-1* mRNA into lighter fractions of the gradient (Figure 4D). This reflects a specific decrease in translational competence of endogenous *glp-1* mRNA as *rpl-11.1*, a germ line-enriched mRNA that encodes a protein of the large ribosomal subunit [39], is unaffected (Figure 4D).

CytoPAPs modify the 3′ends of RNAs [31]. To investigate whether GLD-4 affects the length of the *glp-1* mRNA poly(A) tail, we performed a poly(A) test (PAT) assay [40], and compared endogenous *glp-1* mRNA poly(A) tails, using sucrose gradient fractioned mRNA and non-fractionated input as our starting material. To obtain enough RNA material for the PAT assay and to discriminate translationally active from inactive mRNA pools, we combined several samples of the non-polysomal and polysomal fractions. While all three samples show reduced *glp-1* poly(A) tail lengths in *gld-4*(RNAi) compared to control RNAi knockdowns, we observe no clear difference between the respective non-polysomal and polysomal fractions (Figure 4E,F). The observed poly(A) tail differences are consistent with the contribution of *gld-4* activity to *gld-1* mRNA [40]. This data suggests that GLD-4 cytoPAP activity has an overall impact on *glp-1* poly(A) tail status. Together, our combined results suggest that GLD-4 association with endogenous *glp-1* mRNA may stimulate its efficient translation.

### GLD-4 and GLD-2 cytoPAP expression differs in the PZ

**Figure 5:**
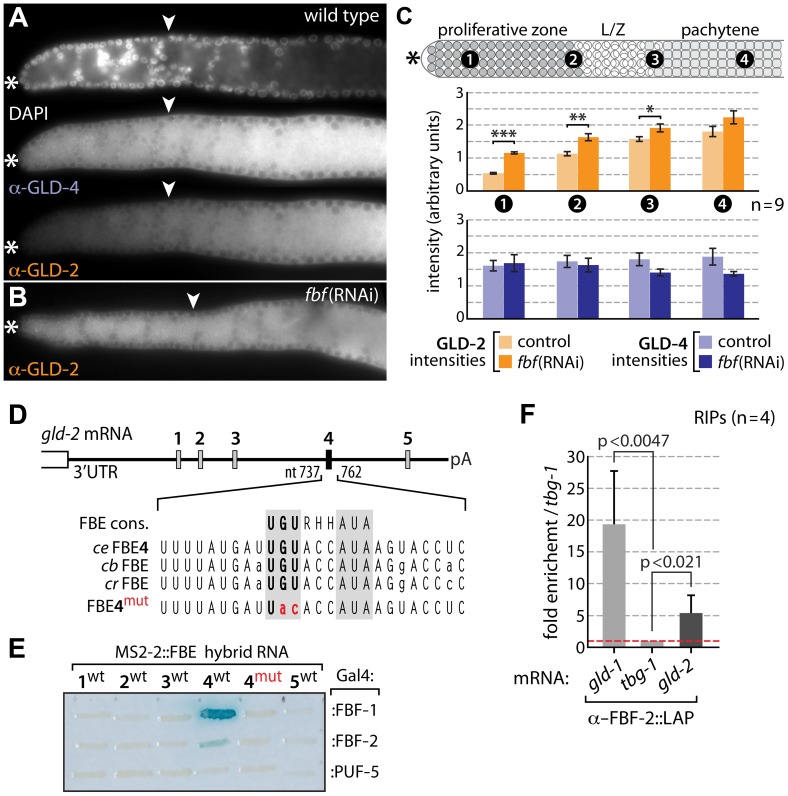
Differential GLD-4 and GLD-2 expression in the proliferative zone is FBF dependent. (A) GLD-4 expression is equal across the distal germ line. GLD-2 intensities increase from low-to-high in a distal-to-proximal manner. Extruded gonads of indicated genotype stained with DAPI, α-GLD-2, α-GLD-4, and α-GLH-2 as a positive tissue penetration control (not shown). Asterisk, distal tip; arrowhead, mitosis-to-meiosis boundary. (B,C) Distal GLD-2 expression is repressed by *fbf* activity. (B) Example of an *fbf*(RNAi) immunostained extruded gonad. For the complete RNAi experiment see Figure S1. (C) Quantification of the complete *fbf*(RNAi) experiment. Four different regions of nine germ lines per genotype were analyzed in their median, primarily cytoplasmic area. Error bars are SEM. ***, p<0.001; **, p<0.01; *, p<0.05; bars without indicated p value are statistically not significant (Student's t-test). (D, E) FBF binds specifically to at least one of the five predicted sequence elements in the *gld-2* 3′UTR. (D) Schematic drawing of the 1094 nt long *gld-2* 3′UTR. Sequence alignment of FBF-binding element consensus (FBE cons.) sequence [14] and the conserved FBE4 element in three *Caenorhabditis* species: *ce*, *C. elegans*; *cb*, *C. briggsae*; *cr*, *C. remanei*. pA indicates beginning of the poly(A) tail. (E) Yeast three-hybrid assay. RNA hybrid and Gal4-protein fusions are indicated. FBF-1, FBF-2 and PUF-5 belong to same RNA-binding protein family. Note, the wild-type (wt) and mutant (mut) sequence of FBE4 tested is larger than the given sequences (see Materials and Methods). A positive and negative control RNA was included (not shown) and protein expression was confirmed by western blotting (not shown). (F) LAP-tagged FBF-2 associates with endogenous *gld-2* mRNA in RNA-coimmunoprecipitation experiments (RIPs) directed against the GFP portion of the fusion protein.

GLD-4 and GLD-2 cytoPAP expression patterns are distinct in “female” germ lines. GLD-4 is expressed equally strong within the entire PZ and in meiosis [30] (Figure 5A). By contrast, GLD-2 is poorly expressed in the distal half of the PZ, becomes more abundant further proximal, and is most abundant in cells that have entered meiosis [28] (Figure 5A). Hence, the differential expression of the two proteins in the PZ may form the basis of GLD-4's unique role in mitosis and GLD-2's role in meiotic entry.

Intriguingly, the protein expression pattern of GLD-2 does not match its ubiquitous mRNA expression pattern in the distal PZ [28]. This suggests translational regulation of GLD-2 expression. An obvious translational repressor in this region is FBF, which represses two mRNAs encoding meiosis-promoting regulators, GLD-1 [18] and GLD-3 [7]. To test for FBF-mediated *gld-2* mRNA repression, we knocked down *fbf* by RNAi and assessed GLD-2 protein abundance in the distal-most germ line by indirect immunofluorescence, using GLD-4 as a reference, and quantified the amounts (Figure 5B,C). While GLD-4 levels are not significantly different between *fbf*(RNAi) and control RNAi germ lines, GLD-2 expression levels in the PZ are higher in *fbf*(RNAi) than in wild type (compare Figure 5A and 5B) or control RNAi experiments (Figure S1A–C). The GLD-2 protein increase is largely limited to the distal half of the PZ: ∼2.2-fold more in cells most distal (Figure 5C, area 1), compared to ∼1.5-fold more in cells most proximal to the PZ (Figure 5C, area 2). Such a restriction to the proliferative zone is consistent with previous reports on FBF activity [18], [20], [41] and suggests that GLD-2 but not GLD-4 is a specific target of FBF regulation.

FBF interacts with mRNAs through the conserved FBF-binding element (FBE) [18]. We identified five putative FBEs in the 3′UTR of *gld-2* mRNA (Figure 5D) and tested each element for binding to FBF protein in a yeast 3-hybrid assay. Only FBE4 in its wild-type sequence was consistently and specifically bound by FBF (Figure 5E). Neither element was bound by PUF-5 (Figure 5E), a different *C. elegans* PUF protein that is abundantly expressed in differentiating female gametes [42]. Intriguingly, the bound FBE sequence is also present in two closely related *Caenorhabditis* species, suggesting that *gld-2* mRNA translational repression may be conserved (Figure 5D). Moreover, RIP experiments of GFP-tagged FBF-2 confirmed a physical association of *gld-2* mRNA with FBF in worm lysates, which appears to correlate with the number of active FBEs in the tested mRNAs (Figure 5F); the positive control, *gld-1* mRNA, possesses two functional FBEs and was enriched strongest [18]. Taken together, we conclude that consistent with published FBF-1 RIP-Chip experiments [19], GLD-2 but not GLD-4 is most likely a direct target of the central mitosis-promoting translational repressor, FBF. Consistent with previous genetic findings [7], an evolutionary conserved translational repression of GLD-2 cytoPAP in undifferentiated cells might be pivotal for the robustness of the balance between proliferation and differentiation.

### The GLD-4/GLS-1 cytoPAP has a second role in promoting meiosis

**Figure 6:**
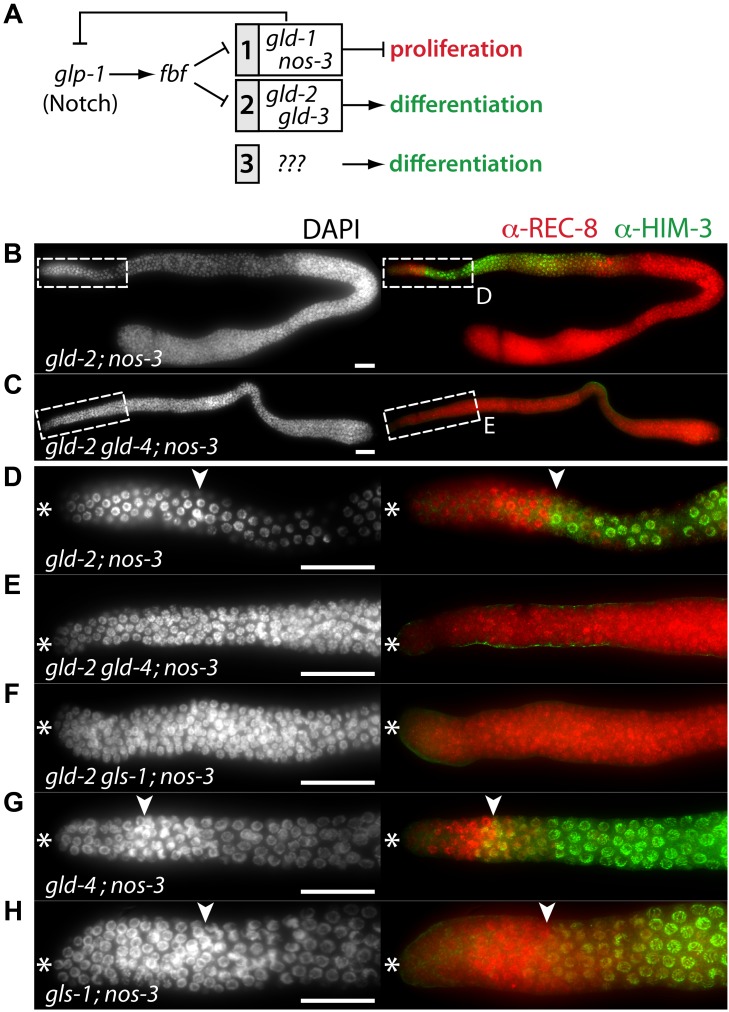
*gld-4* and *gls-1* promote onset of differentiation in parallel to *gld-2* and *nos-3*. (A) The current genetic wiring of the core regulatory network that regulates the balance between proliferation and differentiation onset. Two genetic pathways of redundantly acting translational regulators operate downstream of the translational repressor, FBF. A third, yet undefined, pathway has been evoked [4], [6]. Note that not all genes are equivalent in the two pathways; only *gld-3 nos-3* double mutants lack any signs of differentiation [7]. (B–H) Complete gonads stained with DAPI (left column), and with α-REC-8 and α-HIM-3 (right column) antibodies. Dashed boxes in B and C are close ups of D and E. See Table 1 for the total number of analyzed germ lines. (D–H) Distal region of extruded gonads. Asterisk, distal tip; arrowhead, mitosis-to-meiosis boundary. Scale bars: 50 µm.

The current framework of the core regulatory network underlying meiotic entry appears incomplete and a third meiosis-promoting activity is likely to exist (Figure 6A) [4], [6], [7], [34]. Even though both meiosis-promoting pathways are inactive in the *gld-2; nos-3* double mutant, germ cells enter meiosis [7], [26] (Figure 6B,D; Table 1). Intriguingly, GLD-2 and GLD-4 have a combined function during late meiosis when germ cells are past the onset of differentiation [30]. Hence, it seemed plausible that a further biological overlap of those two enzymes may exist at differentiation onset. Indeed, we find that the triple mutant *gld-2 gld-4; nos-3* lacks any signs of differentiation and it is tumorous (Figure 6C,E; Table 1). This demonstrates that *gld-4* activity promotes meiotic entry in the absence of *gld-2* and *nos-3*.

**Table 1:** Mitosis-to-meiosis decision phenotypes.

strain	genotype	meiotic entry	% entry^1^	n^2^
N2	wild type	yes^3^	100	57
JK3294	*gld-2(q497); nos-3(q650)*	yes^3^	100	38
EV132	*gld-2(q497) gld-4(ef15); nos-3(q650)*	no^3^	0	42
EV373	*gld-2(q497) gls-1(ef8); nos-3(q650)*	no^3^	0	34
EV110	*gld-4(ef15); nos-3(q650)*	yes^3^	100	64
EV80	*gls-1(ef8); nos-3(q650)*	yes^3^	100	45
EV403	*gld-2(q497) gld-1(q485)*	yes^3^	68	119
EV316	*gld-2(q497) gld-1(q485) gld-4(ef15)*	no^3^	0	92
EV318	*gld-2(q497) gld-1(q485) gls-1(ef8)*	no^3^	0	162
EV172	*gld-1(q485) gld-4(ef15)*	yes^3^	100	61
EV178	*gld-1(q485) gls-1(ef8)*	yes^3^	100	135
EV178	*gld-1(q485) gls-1(ef8)*	yes^4^	100	21
EV361	*gld-2(q497) gld-1(q485) gld-4(ef15); glp-1(q175)*	no^3^	0	40
EV351	*gld-2(q497) gld-1(q485) gls-1(ef8); glp-1(q175)*	no^3^	0	78
JK3182	*gld-3(q730) nos-3(q650)*	no^5^	0	21
	*gld-3(q730) nos-3(q650); cye-1*(RNAi)	yes^5^	71	14
EV316	*gld-2(q497) gld-1(q485) gld-4(ef15)*	no^5^	0	24
	*gld-2(q497) gld-1(q485) gld-4(ef15) cye-1*(RNAi)	yes^5^	94	16

1 ) germ lines that contain germ cells with meiotic character.

2 ) number of germ lines.

3 ) assessed by anti-REC-8, anti-HIM-3, and DAPI staining.

4 ) assessed by anti-REC-8, anti-pSUN-1, and DAPI staining.

5 ) assessed by DAPI staining (see Figure 8).

GLS-1 stimulates GLD-4 enzymatic activity and the GLD-4/GLS-1 cytoPAP complex promotes late meiosis [30]. To test if *gld-4* activity requires *gls-1* function for promoting meiotic entry, we generated the *gld-2 gls-1; nos-3* triple mutant. Similar to the *gld-2 gld-4; nos-3* triple mutant, no meiotic entry was observed (Figure 6F; Table 1), indicating a shared function of *gld-4* and *gls-1*. Together this suggests that in addition to a requirement for proliferation, the GLD-4/GLS-1 cytoPAP complex promotes the onset of differentiation in combination with GLD-2 cytoPAP.

A prediction of this model is that the function of a single cytoPAP is enough to promote entry into meiosis in the absence of *nos-3*. Hence, we generated the *gld-4; nos-3* and the *gls-1; nos-3* double mutants. In either double mutant, in comparison to the triple mutant with *gld-2*, we found robust entry into meiosis (Figure 6G,H; Table 1). In conclusion, *gld-4* and *gls-1* promote meiotic entry in parallel to *gld-2* and *nos-3*, suggesting that *gld-4* and *gls-1* might be additional pathway components that promote differentiation onset. Moreover, the striking similarity between the *gld-3 nos-3* double and *gld-2 gld-4; nos-3* triple tumorous germ lines suggest that *gld-2* and *gld-4* or *gls-1* activities are largely equivalent to *gld-3* activity with regard to the meiotic entry process.

### *gld-4* and *gls-1* promote meiosis in parallel to *gld-1* and *gld-2*

**Figure 7:**
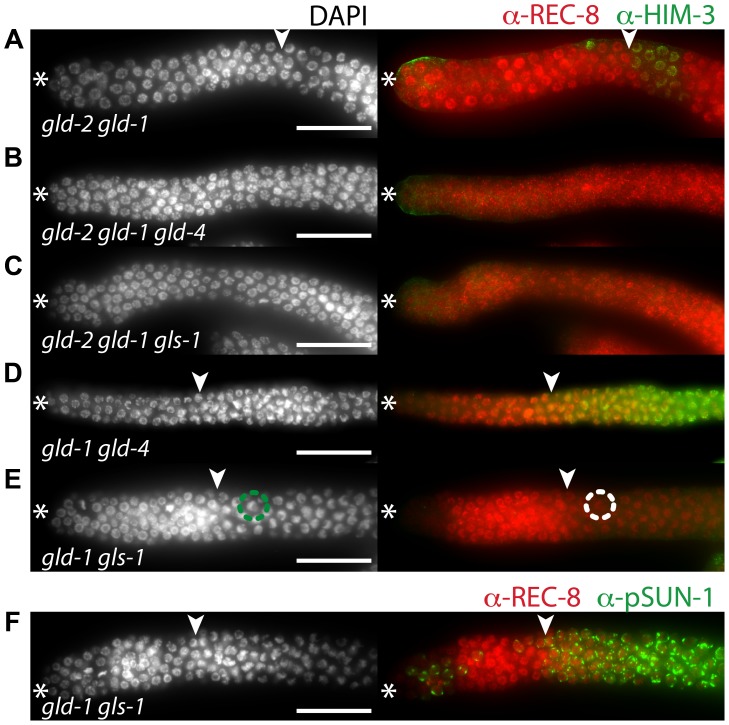
*gld-4* and *gls-1* promote onset of differentiation in parallel to *gld-2* and *gld-1*. (A–F) Distal region of extruded gonads stained with DAPI, and with α-REC-8, α-HIM-3 (A–E), and α-pSUN-1 (F) antibodies. Asterisk, distal tip; arrowhead, mitosis-to-meiosis boundary. Scale bars: 50 µm. See Table 1 for the total number of analyzed germ lines. (E–F) *gld-1 gls-1* double mutant germ lines possess a mitosis-to-meiosis boundary, albeit HIM-3 fails to be detected in E. A strong reduction of nucleoplasmic REC-8, the appearance of crescent-shaped nuclei in meiotic prophase (circles in E), and the abundant expression of pSUN-1 (F) reveal onset of differentiation.

NOS-3 and GLD-1 are assumed to act in a pathway parallel to the GLD-2/GLD-3 cytoPAP pathway (Figure 6A). To complete our analysis of the genetic interactions between the NOS-3/GLD-1 and the GLD-4/GLS-1 cytoPAP pathways, we generated triple mutant strains that had either one of the GLD-4/GLS-1 cytoPAP complex components removed in a *gld-2 gld-1* double mutant background (Figure 7; Figure S2; Table 1).

Germ cells, double mutant for *gld-2 gld-1*, enter meiosis in the majority of germ lines (Figure 7A; Figure S2; Table 1). Germ cells, triple mutant for *gld-2 gld-1 gld-4* (Figure 7B) or *gld-2 gld-1 gls-1* (Figure 7C), failed to enter meiosis and all germ lines are tumorous (Table 1). Importantly, germ cells in the *gld-1 gld-4* (Figure 7D) and the *gld-1 gls-1* (Figure 7E) double mutants enter meiosis (Table 1). Surprisingly, the *gld-1 gls-1* double mutant did not stain for HIM-3 (Figure 7E). However, *gld-1 gls-1* germ cells entered meiosis, as judged by their nuclear architecture, chromosome morphology, and the expression of pSUN-1 (Figure 7F). Our combined results are consistent with the previous triple mutant results, in which a *nos-3* mutant gene replaced *gld-1* (Figure 6), and establish a role of *gld-4* and *gls-1* in the onset of differentiation, suggesting that both genes operate in parallel to *gld-2, gld-1* and *nos-3*.

### Notch activity is dispensable for the proliferation of germ cells that cannot enter meiosis

Notch signaling promotes proliferation, upstream of the meiosis-promoting network [35]. To investigate whether proliferation of tumorous triple mutant *gld-2 gld-1 gld-4* and *gld-2 gld-1 gls-1* germ lines depends on Notch activity, we investigated GLP-1 protein expression and genetically ablated *glp-1* function (Figure S3). In either triple mutant, GLP-1 remains expressed throughout the tumorous germ lines (Figure S3A,C). Consistent with their proliferative activity, dividing cells are scattered throughout the germ line and stain positively for phospho-histone-3 (PH-3) (Figure S3A,C), a marker for cells in prometaphase [43]. Loss of *glp-1* in either triple mutant neither abolishes proliferation nor leads to meiotic entry and cells remain undifferentiated (Figure S3B,D). These results suggest that Notch is not required for proliferation in germ cells that are fully compromised in all meiosis-promoting pathways.

### Proliferation in *gld-2 gld-1 gld-4* tumorous germ lines depends on cyclin E activity

**Figure 8:**
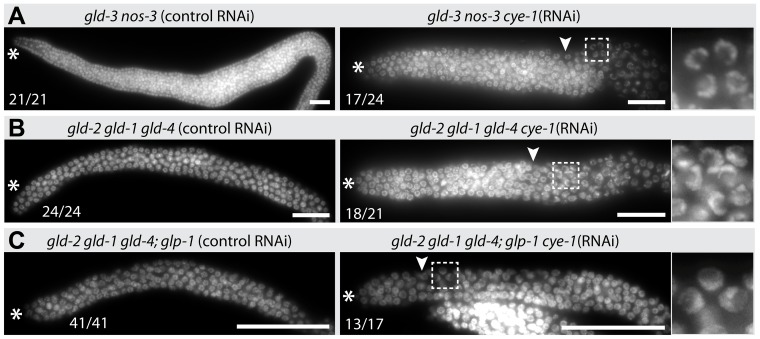
Tumorous proliferation of *gld-2 gld-1 gld-4* triple mutants depends on *cye-1*. (A–C) DAPI staining of extruded distal gonads from indicated genotypes. Control RNAi and *cye-1*(RNAi) was conducted by feeding RNAi bacteria to heterozygote mothers and their homozygote progeny was analyzed 24 hrs past L4. Images of the prevailing phenotype and its occurrence out of all germ lines analyzed are given. The *gld-3 nos-3* tumors served as a positive control for RNAi efficacy. Asterisk, distal tip; arrowhead, mitosis-to-meiosis boundary. Scale bars: 25 µm.

**Figure 9:**
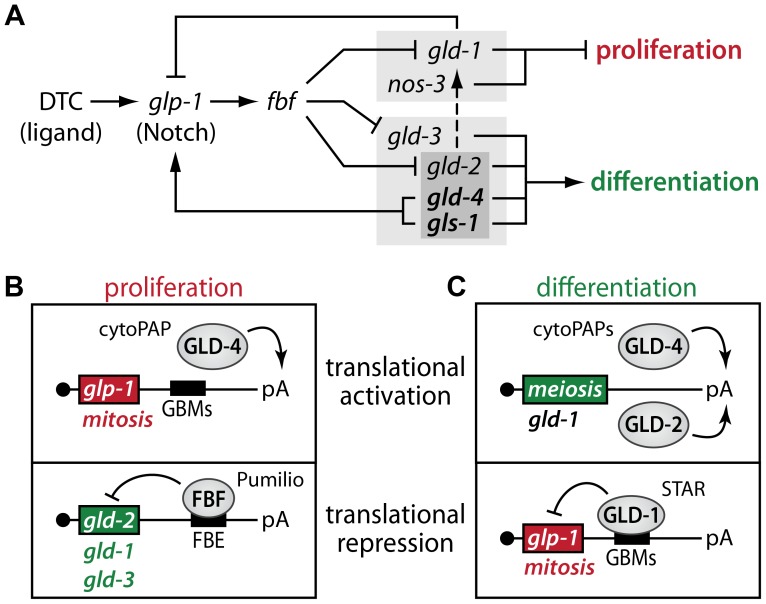
Translational regulators maintain a robust proliferative zone in the adult germ line. (A) Expanded genetic circuitry of primarily translation regulators that fine-tunes the balance between proliferation and differentiation. A light grey box highlights pathway members that regulate differentiation onset. A dark grey box highlights redundant activities that promote GLD-1 expression, primarily when cells commit into meiotic progression (dashed line). See text for details. Note, this simplified circuitry focuses on the RNA regulatory network downstream of GLP-1/Notch and does neither include other known downstream RNA targets nor potential upstream protein regulators. (B) Diagram of translational control examples in proliferative germ cells. Next to *glp-1* mRNA, GLD-4 may also translationally activate additional mRNAs, encoding proliferation-promoting genes. Additional FBF-regulated mRNAs are known that promote the meiotic program. See text for details. (C) Diagram of translational control examples in differentiating germ cells. Additional GLD-regulated mRNAs are known that promote the proliferative fate. See text for details.

Germ cell proliferation in *gld-3 nos-3* tumorous germ lines is independent of Notch signaling but depends on cyclin E [4] (Figure 8A; Table 1). In *cye-1* RNAi knockdown experiments, we found that also *gld-2 gld-1 gld-4* tumorous proliferation requires cyclin E activity (Figure 8B; Table 1). Moreover, consistent with *gld-3 nos-3; glp-1 cye-1*(RNAi) germ lines [4], an additional removal of Notch activity in *gld-2 gld-1 gld-4*; *cye-1*(RNAi) animals increases the ability of germ cells to start meiotic prophase more distally (Figure 8C). In either case, however, differentiation onset is aborted immediately after zygotene/very early pachytene and germ cells do not commit to meiosis. Together, these similarities among the *gld-3 nos-3* and *gld-2 gld-1 gld-4* tumorous germ lines suggest that *gld-4* and, most likely *gls-1*, are components of a meiosis-promoting pathway that acts on the *gld-2* side of both known meiosis-promoting pathways, rather than in a separate, third meiotic entry pathway (summarized in Figure 9) [4].

## Discussion

Our summed findings highlight that translational control, in the combined form of translational activation and repression, serves as a key regulatory mechanism to maintain adult tissue homeostasis in the *C. elegans* germ line (Figure 9). Central to our findings is the dual activity of the GLD-4/GLS-1 cytoPAP complex, which has a major role in promoting germ cell proliferation and a minor role in differentiation onset. By focusing on the activity of numerous key RNA regulators, this work expands the known core genetic circuitry downstream of niche-mediated Notch signaling that governs the balance between proliferation and differentiation (summarized in Figure 9A). At the molecular level, we propose a rheostat that consists of two translational control modules, one specific for proliferation (Figure 9B) and one specific for differentiation onset (Figure 9C). Both modules are interconnected via their mRNA targets, and this reciprocal translational activation and repression of either proliferation or differentiation factors may fine-tune the size of the proliferative zone.

### GLD-4 cytoPAP and FBF function to maintain the proliferative zone

The GLD-4/GLS-1 cytoPAP complex has multiple roles in germ cell development [30], [33]. In this work, we demonstrate that both complex members contribute to the maintenance of the size of the proliferative zone by primarily influencing adult germline proliferation and secondarily differentiation onset. This dual role is consistent with the ubiquitous expression of both proteins in the respective regions of the adult germline tissue [30], [33]. GLD-4 is the enzymatic component of the GLD-4/GLS-1 cytoPAP complex and is evolutionarily most similar to nuclear Trf4/5-type polymerases, which add short poly(A) tails to nonproductive RNA molecules to initiate exosome-mediated RNA degradation [32]. By contrast, GLD-4 and its enzymatic activator GLS-1 are cytoplasmic proteins implicated in translational control [30], [33]. The notion that translational activation of mRNAs is coupled to cytoplasmic poly(A) tail extension or maintenance is primarily shaped by the work on poly(A) polymerases, such as members of the conserved GLD-2 family [31]. By analogy, GLD-4 cytoPAP's role in proliferative germ cells may therefore translationally activate mitotic-fate promoting mRNAs. We provided four pieces of evidence that the Notch receptor-encoding *glp-1* mRNA is a likely mRNA target of GLD-4/GLS-1 cytoPAP activity: (1) GLD-4 associates with *glp-1* mRNA, and (2) positively influences its poly(A) tail length. (3) Furthermore, we found that expression of a *glp-1* 3′UTR translational reporter and that of endogenous GLP-1 protein depends on GLD-4 presence. (4) Lastly, translational efficiency of endogenous *glp-1* mRNA requires *gld-4* activity. Therefore, these results combined support the idea that abundant GLP-1 expression is maintained by GLD-4-mediated translational activation. However, the partial reduction in *glp-1* poly(A) tail length might either reflect an intrinsic enzymatic difference between Trf4-type PAPs and GLD-2, or suggests that other, yet undiscovered cytoPAPs may work redundantly to GLD-4. Alternatively, additional poly(A) tail-independent mechanisms for GLD-4-mediated translational activation may exist. Regardless of the precise molecular function of GLD-4, the translational repressor of *glp-1* mRNA, GLD-1 protein, starts to accumulate in the proximal part of the PZ, prior to the mitosis-to-meiosis boundary [44], suggesting that *glp-1* mRNA may already be subject to translational repression in the proximal PZ. Therefore, to ensure robust GLP-1 protein expression, GLD-4-mediated translational activation of *glp-1* mRNA may help to counteract GLD-1-mediated translational repression to maintain the size of the PZ in the adult (Figure 9A,B). However, *glp-1* mRNA is presumably not the only target of the GLD-4/GLS-1 cytoPAP complex, and others are likely to exist.

In the balance between proliferation and differentiation, the two translational activators, GLD-4 and GLD-2, seem to have antagonistic roles that may also constrain their regulation and function in the PZ. A loss of GLD-4 shrinks the PZ and a loss of GLD-2 expands the PZ. Therefore, GLD-2 may promote meiosis at the expense of mitosis in the *gld-4* single mutant. Conversely, GLD-4 may be responsible for the expansion of the PZ in the *gld-2* single mutant. Importantly, upon loss of both cytoPAP activities, the PZ re-adjusts to an intermediate size, arguing that they form an antagonistic pair. In particular, the distinct expression profile of either cytoPAP presumably reflects and affects their divergent roles in regulating mRNA-specific gene expression. The delay of GLD-2 protein expression in the PZ correlates with its genetic requirement for the onset of differentiation and a putatively required absence in undifferentiated cells. Moreover, its 2–3 fold lower abundance in the distal half of the PZ may selectively favor and functionally constrain GLD-4-mediated germ cell proliferation. Hence, a healthy balance between GLD-2 and GLD-4 functions appears to be perpetuated to maintain the size of the adult PZ.

To maintain adult germ cell proliferation and prevent progressive shrinkage of the PZ, *gld-2* mRNA translation is delayed by FBF, a dominant translational repressor of several meiosis-promoting genes [7], [18], [19], [45]. We found that GLD-2 but not GLD-4 cytoPAP accumulation in the PZ appears to be inhibited by FBF, and that *gld-2* mRNA associates with FBF most likely at least through one FBF-binding site in its 3′UTR. Therefore, a translational repressor (FBF) that turns off the activities of mRNAs encoding meiosis-promoting proteins (e.g. GLD-2) is combined with a translational activator (GLD-4) that turns on mRNA activities that encode mitosis-promoting proteins (e.g. GLP-1) to maintain germ cell proliferation (Figure 9A,B).

### GLD-4 and GLD-2 cytoPAP stimulate entry into meiotic prophase

Conversely to germ cell proliferation, the onset of differentiation requires translational repressors (GLD-1 and NOS-3) that presumably turn off mRNA activities encoding mitosis-promoting proteins and translational activators (GLD-2, GLD-3, GLD-4, and GLS-1) that presumably turn on mRNA activities encoding meiosis-promoting proteins [6], [7], [26], [35] (Figure 9A,C). Previous genetic work established two parallel pathways, which either indirectly or directly promote differentiation onset (Figure 6A). However, not all components are equal in their potential to contribute to meiotic prophase entry. In this regard, the synergism of NOS-3 and GLD-3 is of equal strength, as is NOS-3 with both GLD-2 and GLD-4/GLS-1, or, GLD-1 with both GLD-2 and GLD-4/GLS-1. Hence, our findings of a dual role for GLD-4 cytoPAP strengthens the role of translational control even further, highlights the importance of translational activation for the balance of proliferation and differentiation, and clarifies the many levels of redundancy within the two, major, parallel pathways of the current genetic circuitry (Figure 9A).

Differentiation onset deploys two translational activators of presumed meiosis-promoting mRNAs (Figure 9C). In this regard, GLD-2 cytoPAP performs a more prevalent role in activating meiosis-promoting mRNAs as its combined loss with genes of the first, translational repressor pathway (*i.e. gld-1 gld-2* or *gld-2; nos-3* doubles) causes more germ cell overproliferation than is observed in the respective *gld-4* double mutant germ lines. Importantly, germ cells of *gld-2*; *nos-3* or *gld-2 gld-1* double mutants enter meiosis in a *gld-4-* and *gls-1*-dependent manner, as triple mutant germ cells (e.g. *gld-1 gld-2 gld-4* or *gld-2 gld-4; nos-3*) do not enter meiosis. Consistent with previous findings that germline proliferation in tumorous *gld-2 gld*-*1* or *gld-3 nos-3* double mutants is *glp-1*-independent [4], [35], tumorous triple mutant *gld-2 nos-3* germ cells that lack in addition either *gld-4* or *gls-1* do not require GLP-1 activity to remain in mitosis either, arguing for their genetic position downstream of Notch and in parallel to each other for meiotic entry (Figure 9A). Intriguingly, the similarities between the *gld-3 nos-3* double and *gld-2 gld-4; nos-3* or *gld-2 gls-1; nos-3* triple mutants suggest further that *gld-3* activity equals the combined activities of *gld-2* and *gld-4/gls-1* with respect to the loss of *nos-3*, which positions *gld-4/gls-1* within the second, translational activator pathway at the level of *gld-2* (Figure 9A). These genetic behaviors appear to parallel the known molecular protein interactions. The multi-KH domain protein GLD-3 binds directly to GLD-2 cytoPAP and GLS-1 [30], [33], illustrating that GLD-3 may serve as an integral regulatory factor for both GLD-2 and GLD-4/GLS-1 cytoPAPs to promote differentiation onset.

Redundancy of cytoPAP-mediated translational activation has been previously reported in a later step of meiotic prophase of female germ cells that require abundant GLD-1 expression for meiotic commitment [30]. Intriguingly, *gld-2 gld-4* double mutant germ cells enter meiosis [30], suggesting that the remaining low GLD-1 amounts might be sufficient to promote meiotic entry. Consistent with this idea, *gld-2 gld-4 gld-1* triple mutant germ cells never enter meiosis, arguing that in the absence of cytoPAP activity, the remaining *gld-1* activity/GLD-1 amount is indeed crucial for meiotic entry. In agreement with previous findings [4], [46], [47], our work suggests that for differentiation onset in *gld-2 gld-4* double mutants, cyclin E represents an important target of GLD-1-mediated translational repression. However, we expect additional differentiation onset-promoting mRNA targets to be positively regulated by GLD-2 and GLD-4, either in a combinatorial manner or separately. Alternatively, other RNA-directed molecular functions, such as miRNA stability described for GLD-2 orthologs in mammals [48], might be relevant. Future research on the RNA-regulatory repertoire of GLD-2 and GLD-4 will be required to better resolve these issues.

We propose that two modules of translational activation and repression, interconnected via their mRNA targets, establish a molecular rheostat that leads to a reciprocal expression of either proliferation or differentiation factors. Together they maintain adult germline proliferation in adult *C. elegans* animals. Translational repression, in particular, is an established mechanism in *Drosophila* and *C. elegans* development. Translational control is also an essential mechanism of the transition from self-renewal/proliferation to differentiation in *Drosophila* germ cells [49], [50]. Our work suggests that the regulation of turning translation on is equally important for maintaining a healthy balance between proliferation and differentiation as turning translation off. With this work, we begin to fill this obvious gap in our understanding of adult tissue maintenance.

## Materials and Methods

### Strains and RNAi knockdown

*C. elegans* strains were handled according to standard procedures [51]. Worms were grown at 20°C and used for most experiments at an age of 24 hours (h) past mid-L4. Bristol N2 served as the wild-type strain.

Mutations used: LGI: *gld-2(q497)*, *gld-1(q485)*, *fer-1(b232)*, *gls-1(ef4)*, *gls-1(ef8)*, *gld-4(ef9)*, *gld-4(ef15)*. LGII: *gld-3(q730), nos-3(q650)*. LGIII: *glp-1(q175)*. Transgenes used: *rrrSi117[Pmex-5*::*GFP::H2B::glp-1(wt 3′UTR) unc-119(+)] II*, *rrrSi118[Pmex-5::GFP::H2B::glp-1(GBM1,2,3 mut 3′UTR) unc-119(+)] II*; both are Mos1-mediated single copy gene insertions and their sequences are described in the supplemental text of [25]. JH2929 expresses the LAP-tagged FBF-2 [52].To generate the new *gld-2(q497) gld-1(q485)* double mutant, we crossed heterozygous *gld-2* males with heterozygous *gld-1* hermaphrodites. Next, we crossed F1 non-green worms, containing *gld-1* and *gld-2*, and green siblings, containing the *hT2[qIs48] I;III* balancer. In the F2 progeny heterozygous balancer animals were screened for a recombination event between the *gld-2* and the *gld-1* locus by genomic PCR for the *q485* deletion and sequencing for the *q497* point mutation. Homozygote *hT2[qIs48] I;III* animals are embryonic lethal and cannot be analyzed as a wild-type sibling control.

All other double and triple mutants on LGI were generated in a similar manner and balanced by *hT2[qIs48] I;III* and are listed in Table 1. Quadruple mutants containing genes on LGI and LGIII were balanced by *hT2[qIs48] I;III* and validated by PCR for deletions and by sequencing for *gld-2(q497)* and *glp1(q175)*. Double and triple mutant combinations on LGI and LGII were maintained by a closely linked GFP transgene *(ccIs4251)* to *unc-15(e73)* on LGI and *mIn1[mIs14 dpy-10(e128)]* on LGII. The presence of all mutations was validated by PCR for deletions or by sequencing. Primer sequences are available on request.

RNAi experiments were performed according to published feeding RNAi procedures [53]. The *fbf* RNAi construct corresponds to *fbf-1* (nts 1040–1845), *cye-1* is described elsewhere [54], and the empty pL4440 vector served as a control. L4-staged N2 animals were placed on RNAi plates and analyzed 24 h later. The efficiency of *fbf* knockdown was confirmed by a loss of anti-FBF immunoreactivity 24 h past L4, and after continued feeding at 48 h past the L4 stage by phenotypic changes of the germ lines, *i.e.* the shrinkage of the PZ and even later the appearance of male-fated germ cells [55].

### Immunocytochemistry and immunoblotting

Antibodies against the following proteins were used as described: anti-HIM-3 1∶200 [56], anti-pSUN-1 1∶1000 [57], anti-GLD-4 1∶20 [30], anti-GLP-1 1∶10 [13], anti-PH-3 1∶500 (9706, Ser-10, 6G3, Cell Signaling), anti-FBF-1 1∶100 [55], anti-GLH-2 1∶200 [58], anti-GLS-1 1∶20 [33]. Monoclonal anti-REC-8 1∶20 (mo560-G25-1, at 10 ng/ul) and anti-GLD-2 1∶20 (A4-4, at 10 ng/ul) antibodies were generated against recombinant HIS-REC-8(aa330–525) and GST-GLD-2(aa959–1113) fusion peptides. The antibodies are specific to the respective proteins; no immunocytochemistry signal was observed in corresponding null mutants and the protein expression patterns in wild type match published ones [28], [59]. Secondary antibodies (1∶500) were coupled to FITC, CY3 and CY5 (Jackson Labs).

Extruded germ lines were prepared in solution as described [33]. The correct localization and comparable intensities of GLH-2 served as a tissue penetration control for all immunofluorescence experiments. Images were acquired with Axiovision Software (Zeiss) on a wide-filed Imager Z1 (Zeiss) microscope, equipped with an AxioCam MRm (Zeiss) camera. Raw images were processed in Photoshop CS5 (Adobe) and assembled in Illustrator CS5 (Adobe). For quantification of immunofluorescent intensities, all images for comparison were taken with identical settings. A median focal plane was chosen where the syncytium was at its maximum width. The pixel intensities were measured in Fiji (ImageJ). To compare GLP-1 intensities, a line scan was performed as is indicated in Figure 3B, ranging from the distal germline tip to the beginning of pachytene. Then all values were binned into the 10 fractions whose positions are displayed in Figure 3B. Averages of those fractions between all analyzed germ lines were calculated and normalized to GLH-2 intensities (measured in the same way). To compare cytoplasmic GLD-2 and GLD-4 intensities, four identical circles were placed over the rachis of the distal arm along the distal-to-proximal axis as indicated in Figure 5C (five germ cell diameters (GCD) proximal of the distal tip, at the end of the PZ, at the beginning of pachytene, and ten GCD into pachytene) and averaged for all germ lines per genotype. The GLD-2/GLD-4 intensities given are not normalized to the GLH-2 signals, which were in these cytoplasmic regions very low. To ensure equal penetration, we independently measured the peri-nuclear GLH-2 signal in neighboring nuclei and found it very similar among all analyzed germ lines.

Immunoblots were performed according to standard procedures with a mixture of two anti-GFP antibodies, at a final dilution of 1∶1000 (11814460001, clones 7.1 and 13.1, Roche) and 1∶200 (sc-9996, B-2, Santa Cruz), anti-tubulin 1∶100000 (T 5168, clone B-5-1-2, Sigma), and HRP-conjugated anti-mouse secondary antibodies (Jackson Labs).

### Yeast three-hybrid

Three-hybrid experiments were performed as described [60]. *gld-2* RNA sequences were cloned into the Xma*I* and Sph*I* sites of the vector pIIIA/MS2-2, using either PCR-amplified fragment (FBE4) or annealed synthetic oligonucleotides (remaining FBE sites). Their nucleotide positions in relation to the first nucleotide of the *gld-2* 3′UTR are as follows: FBE1 (nts 298–335); FBE2 (nts 354–394); FBE3 (nts 460–493); FBF4 (nts 683–763); FBE5 (nts 952–986). For binding specificity, a mutation (UG to AC, see Figure 4D) was engineered by site-directed mutagenesis using Quikchange (Stratagene).

### mRNA analysis

For the sucrose gradient experiments, whole-worm extracts of L1 synchronized adult animals (L4+24 h) grown in comparable feeding-RNAi conditions were prepared by pulverizing frozen worms and adding lysis buffer [50 mM HEPES pH 7.5, 125 mM KCl, 5 mM MgCl2, 1 mM DTT, 0.005% NP-40, 2× Protease Inhibitor Cocktail without EDTA (Roche), 100 U/ml Ribolock (Fermentas), 2 mM PMSF, 4 mM Benzamidine, 2 µg/ml Leupeptin, 2 µg/ml Pepstatin, 0.1 µg/ml Pefabloc, 2 mM NaF, 2 mM Na3VO3 and 200 µg/ml Cycloheximide], followed by a low speed centrifugation to removed insoluble components. The clear supernatant of three biological replicates was layered onto a 17–50% w/v sucrose gradient and processed as previously described [61] with the only exception that the gradients were spun for 210 min. For the mRNA distributions analysis, 10 fmole of a polyadenylated *in vitro* transcribed luciferase mRNA was added to each fraction prior to RNA isolation as an internal RNA standard for extraction efficiency. The Trizol (Invitrogen) isolated RNA from individual fractions was resolved in equal volumes of water and further analyzed by qRT-PCR or pooled for splint-mediated poly(A) tests [40].

RIP experiments were performed from mixed-stage animals as described [62] using anti-GFP (MPI-CBG), anti-GLD-4 [30], or anti-GLS-1 [33] antibodies. Semiquantitative RT-PCR samples of three independent RIP experiments were resolved on ethidium bromide-stained agarose gels. The control samples without Reverse transcriptase were negative and are not shown. For the qRT-PCR experiments, equal volumes of gradient fractionated RNA, total RNA, or RIP material of three biological replicates was used as input material. cDNA synthesis was performed using Revert Aid Premium Transcriptase (Fermentas) in combination with oligo dT primers, following the manufactures protocol. qPCRs were performed on a Mx3000P qPCR system (Stratagene) using ABsolute qPCR SYBR Green mix (Thermo) under standard conditions.

For measuring poly(A) tail length, we pooled five non-polysome and polysome fractions as indicated in Figure 4. Together with 4 µg total RNA of the input material, we processed the pooled sucrose gradient experiments by ligating an RNA anchor to the 3′ends, preformed a gene-specific RT-PCR, and resolved the DNA samples on a high-resolution agarose gel, according to [40]. Lane quantifications were performed using Fiji (ImageJ), as described in [40].

## Supporting Information

Figure S1 Differential GLD-4 and GLD-2 expression in the proliferative zone is dependent on *fbf*. (A) GLD-2 intensities increase in the distal germ line. Extruded hermaphrodite gonads of control and *fbf*(RNAi) animals stained with α-GLD-2 antibody. (B) GLD-4 intensities remain similar in the distal germ lines shown in (A). (C) Quantification of germ lines with increased GLD-2 levels in RNAi-treated animals. (A,B) Asterisk, distal tip; arrowhead, mitosis-to-meiosis boundary. Scale bar: 25 µm.
(TIF)

Figure S2 *gld-1 gld-2* double mutant germ cells enter meiotic prophase. Extruded gonad of a newly created *gld-1 gld-2* double mutant strain (see experimental procedures) stained with DAPI (top), α-REC-8 and α-HIM-3 antibodies (bottom). Asterisk, distal tip; arrowhead, mitosis-to-meiosis boundary. Scale bar: 25 µm.
(TIF)

Figure S3 Tumorous proliferation of *gld-2 gld-1 gld-4* triple mutants is independent of Notch activity. (A-D) Distal region of immunostained extruded gonads. Asterisk, distal tip; arrowhead, mitosis-to-meiosis boundary. Scale bars: 50 µm. (A,C) Germ line tumors express ubiquitously the GLP-1/Notch receptor and possess stochastically nuclei in mitotic prometaphase (α-Phospho-Histone-3, PH-3). (B,D) Mitotic activity in tumorous germ lines is *glp-1* independent. White dashed lines, distal gonads.
(TIF)
