# Correction: GLD-4-Mediated Translational Activation Regulates the Size of the Proliferative Germ Cell Pool in the Adult *C*. *elegans* Germ Line

**DOI:** 10.1371/journal.pgen.1005862

**Published:** 2016-02-09

**Authors:** Sophia Millonigg, Ryuji Minasaki, Marco Nousch, Jakub Novak, Christian R. Eckmann

Jakub Novak should be included in the author byline instead of the Acknowledgments. He should be the fourth author, and his affiliation is: Max Planck Institute of Molecular Cell Biology and Genetics (MPI-CBG), Dresden, Germany

The correct citation is: Millonigg S, Minasaki R, Nousch M, Novak J, Eckmann CR (2014) GLD-4-Mediated Translational Activation Regulates the Size of the Proliferative Germ Cell Pool in the Adult *C. elegans* Germ Line. PLoS Genet 10(9): e1004647. doi:10.1371/journal.pgen.1004647

The correct author contributions are:

Conceived and designed the experiments: SM CRE. Performed the experiments: SM RM JN MN CRE. Analyzed the data: SM RM MN CRE. Contributed reagents/materials/analysis tools: SM RM MN CRE. Wrote the manuscript: SM CRE.

The correct Acknowledgments are: We are thankful to Jane Wright and Rafal Ciosk (FMI) for generously providing the transgenic lines; David Rudel, Sarah Crittenden, Carrie Cowan, and Eli Knust for critically reading the manuscript; Mark Schmid and Agata Rybarska for initial strain analysis; Carrie Cowan for the cye-1(RNAi) construct; the MPI-CBG antibody and protein expression facilities for antisera and technical support concerning gradient fractionations, respectively; Judith Kimble and Verena Jantsch-Plunger for antibodies; the CGC nematode stock center for providing strains.

The authors confirm that the addition of this co-author does not affect the competing interest statement or funding statement.
