# Supplementary figures and images for: GLD-4-Mediated Translational Activation Regulates the Size of the Proliferative Germ Cell Pool in the Adult C. elegans Germ Line
Source: PLoS Genet. 2014 Sep 25;10(9):e1004647. doi: 10.1371/journal.pgen.1004647 (PMC4177745; doi:10.1371/journal.pgen.1004647)

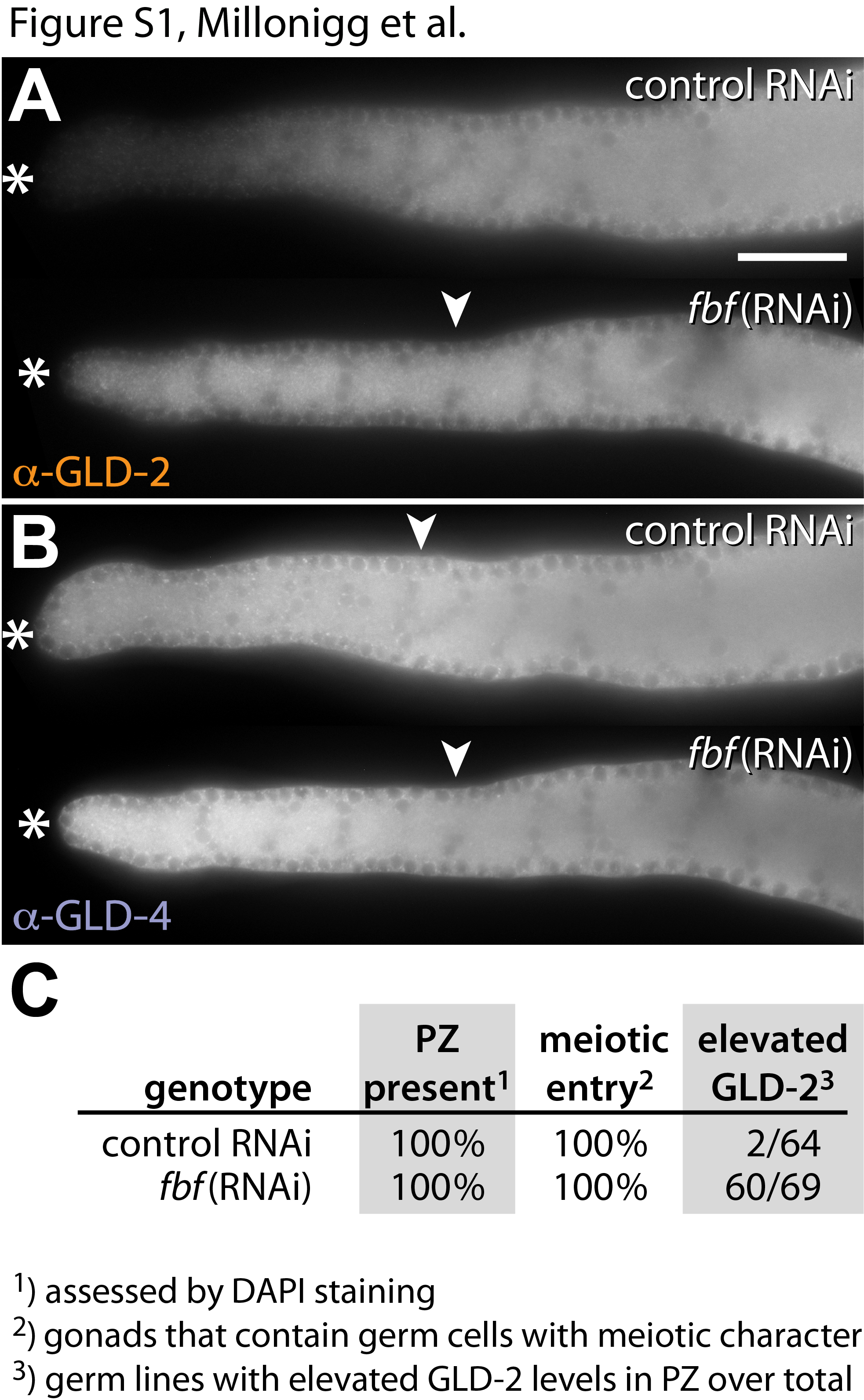

Supplement: Figure S1 — Differential GLD-4 and GLD-2 expression in the proliferative zone is dependent on fbf. (A) GLD-2 intensities increase in the distal germ line. Extruded hermaphrodite gonads of control and fbf(RNAi) animals stained with α-GLD-2 antibody. (B) GLD-4 intensities remain similar in the distal germ lines shown in (A). (C) Quantification of germ lines with increased GLD-2 levels in RNAi-treated animals. (A,B) Asterisk, distal tip; arrowhead, mitosis-to-meiosis boundary. Scale bar: 25 µm. (TIF) [file pgen.1004647.s001.tif]

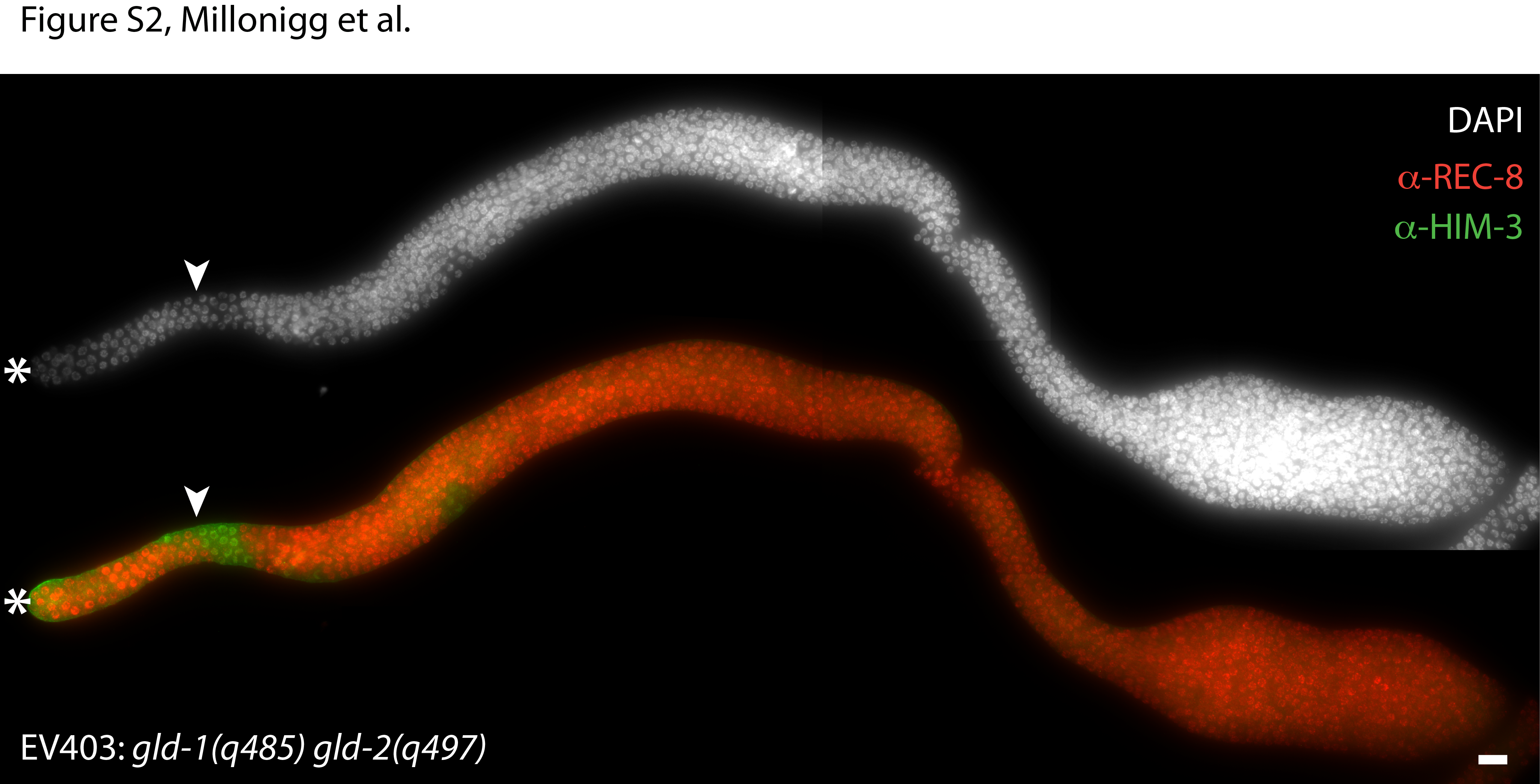

Supplement: Figure S2 — gld-1 gld-2 double mutant germ cells enter meiotic prophase. Extruded gonad of a newly created gld-1 gld-2 double mutant strain (see experimental procedures) stained with DAPI (top), α-REC-8 and α-HIM-3 antibodies (bottom). Asterisk, distal tip; arrowhead, mitosis-to-meiosis boundary. Scale bar: 25 µm. (TIF) [file pgen.1004647.s002.tif]

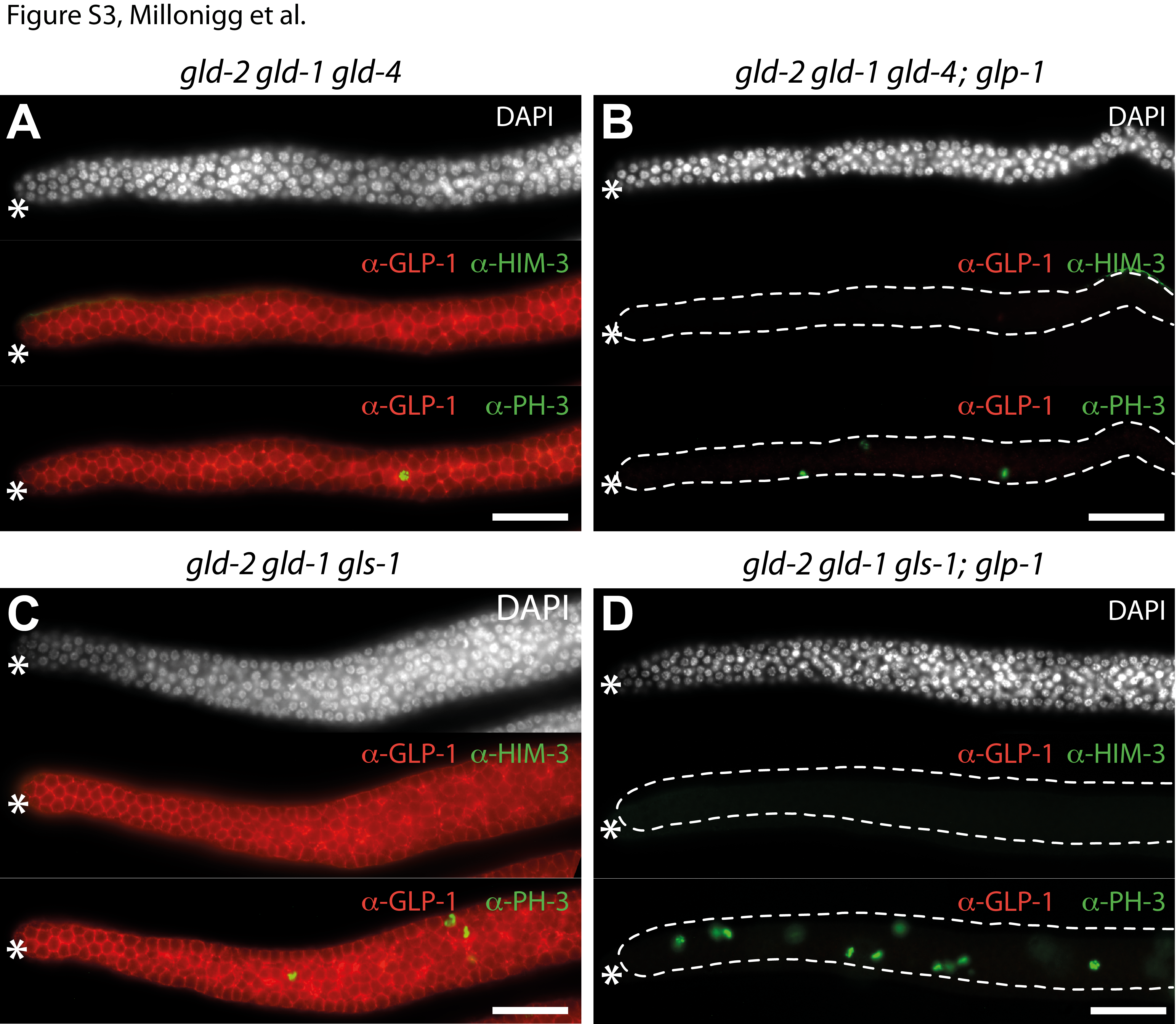

Supplement: Figure S3 — Tumorous proliferation of gld-2 gld-1 gld-4 triple mutants is independent of Notch activity. (A-D) Distal region of immunostained extruded gonads. Asterisk, distal tip; arrowhead, mitosis-to-meiosis boundary. Scale bars: 50 µm. (A,C) Germ line tumors express ubiquitously the GLP-1/Notch receptor and possess stochastically nuclei in mitotic prometaphase (α-Phospho-Histone-3, PH-3). (B,D) Mitotic activity in tumorous germ lines is glp-1 independent. White dashed lines, distal gonads. (TIF) [file pgen.1004647.s003.tif]
